# The Application of an Implementation Science Framework to Comprehensive School Physical Activity Programs: Be a Champion!

**DOI:** 10.3389/fpubh.2017.00354

**Published:** 2018-01-05

**Authors:** Justin B. Moore, Russell L. Carson, Collin A. Webster, Camelia R. Singletary, Darla M. Castelli, Russell R. Pate, Michael W. Beets, Aaron Beighle

**Affiliations:** ^1^Department of Family and Community Medicine, Wake Forest School of Medicine, Winston-Salem, NC, United States; ^2^Department of Implementation Science, Wake Forest School of Medicine, Winston-Salem, NC, United States; ^3^School of Sport and Exercise Science & Colorado School of Public Health, University of Northern Colorado, Greeley, CO, United States; ^4^Department of Physical Education, University of South Carolina, Columbia, SC, United States; ^5^Department of Kinesiology and Health Education, College of Education, University of Texas at Austin, Austin, TX, United States; ^6^Department of Exercise Science, Arnold School of Public Health, University of South Carolina, Columbia, SC, United States; ^7^Department of Kinesiology and Health Promotion, College of Education, University of Kentucky, Lexington, KY, United States

**Keywords:** school physical activity promotion, physical education teachers, community-engaged research, communities of practice, service-learning

## Abstract

Comprehensive school physical activity programs (CSPAPs) have been endorsed as a promising strategy to increase youth physical activity (PA) in school settings. A CSPAP is a five-component approach, which includes opportunities before, during, and after school for PA. Extensive resources are available to public health practitioners and school officials regarding *what* should be implemented, but little guidance and few resources are available regarding *how* to effectively implement a CSPAP. Implementation science provides a number of conceptual frameworks that can guide implementation of a CSPAP, but few published studies have employed an implementation science framework to a CSPAP. Therefore, we developed Be a Champion! (BAC), which represents a synthesis of implementation science strategies, modified for application to CSPAPs implementation in schools while allowing for local tailoring of the approach. This article describes BAC while providing examples from the implementation of a CSPAP in three rural elementary schools.

## Introduction

Youth across the United States are insufficiently physically active (1). To address this public health crisis, numerous physical activity (PA) interventions have been developed and tested within the community setting (2). Unfortunately, these have resulted in limited impact on children’s PA. A recent meta-analysis on randomized-controlled trials using accelerometry (i.e., objectively measured PA) as the outcome measure concluded that “*physical activity interventions for children have little effect on overall activity levels*” (3). Despite these discouraging findings, comprehensive school physical activity programs (CSPAPs) have been nationally endorsed as a promising strategy to increase youth PA through schools (4). A CSPAP is a five-component approach to PA promotion to include PA opportunities before, during, and after school, with the goal of youth accumulating 60 min of PA per day (5). CSPAP activities are typically coordinated at the school level by a “Champion” [also called a “Physical Activity Leader” (6) or “Director of Physical Activity” (7)] who is tasked with leading implementation efforts (7). A recent systematic review and meta-analysis of PA interventions in schools (8) indicated that few interventions target multiple components of the CSPAP model, and the overall effect of the interventions on youth PA was minimal. However, the more components that were targeted, the more effective the interventions were, with multicomponent approaches most promising. This relative lack of effectiveness has been confirmed in recent work, a modest arrest in longitudinal declines recorded in youth PA with 2 min more of moderate-to-vigorous PA (MVPA) levels (15 vs. 13 min) at 8 months following intervention initiation (9). However, more substantial CSPAP impacts have been demonstrated at 4 months post-intervention, with to 5 min more MVPA and 6.5 more shuttle runs (PACER) in a separate study (10). Unfortunately, a recent review indicated that few existing studies link implementation to outcomes, suggesting that implementation monitoring is severely lacking (11). These findings suggest that the literature is missing two important concepts; (a) guidance and examples to inform implementation monitoring to establish the relationship between implementation fidelity and/or dose and PA in affected youth and (b) frameworks that could be utilized to improve the implementation and effectiveness of CSPAPs. This article is an attempt to address these gaps in the literature, as we present the application of an evidence-based implementation framework to the adoption of a CSPAP in elementary school settings in the form of Be a Champion!

## Comprehensive School Physical Activity Programs

Guided by early and recent coordinated models to school health (12, 13), CSPAP is a whole-of-school approach for improving youth PA behaviors. The CSPAP model is based on two core premises: (1) schools are logical sites for influencing youth PA levels as most children spend the majority of their waking hours in/around school (14), (2) physical education curricula and its importance to developing physically active lifestyles are the cornerstone of CSPAP, but rarely offered enough to be the sole emphasis (15). As such, youth have better chance of meeting the daily 60-min PA recommendation though the coordinated provision of five CSPAP components: *quality physical education* classes as the foundation (e.g., emphasizing knowledge, skills, and confidence to move for a lifetime), *PA during school* (e.g., recess and classroom movement activities), *before and after school programs* (e.g., active transportation and activity clubs), *staff involvement* (e.g., employee wellness programs with PA as a priority outcome), and *family and community engagement* (e.g., family activity outings, school site as community center for PA). CSPAP interventions often focus on maximizing school movement experiences by expanding, extending, or enhancing existing opportunities across the CSPAP components (16). We feel that support for CSPAPs has steadily grown with efforts such as former First Lady Michelle Obama’s *Let’s Move!* Active Schools campaign,^1^ and a recent, stand-alone CSPAP webpage^2^ supported by a growing collaboration of organizational partners and school change agents.

## Implementation Science

Implementation science has evolved as a field over the last 50 years, evolving from economic and social programs of 1960s America that required uptake, adoption, and fidelity to policies targeting population ills such as poverty (17). The field evolved further with the launch of Healthy People in 1979 and subsequent efforts to measure progress toward the stated objectives (18). At this stage, much of what was to become implementation science was focused on quality improvement in delivery of clinical services. Subsequent iterations, starting with Healthy People 2000, were launched with measures and implementation strategies. As these strategies continued to evolve, a greater focus on translation of evidence-based practices (EBPs) into real-world settings (e.g., hospitals, clinics, health departments, and schools) has emerged. This translational focus has encouraged acknowledgment of the complex and dynamic nature of the systems in which the interventions are to be delivered, and the inconsistencies between the goals of those wishing to implement new strategies and the community members expected to take up the innovative practices (19, 20). We believe that schools are an excellent example of a complex and dynamic system, and CSPAP implementation is often perceived as inconsistent with the goals of teachers and administrators.

## Implementation Frameworks

There are a large number of implementation frameworks that have been developed to guide implementation efforts generally, and in very specific settings. A recent review by Tabak et al. (21) included 61 models suggests that they share a number of common attributes, and work by Meyer and colleagues to synthesize the literature resulted in the Quality Implementation Framework (QIF) (22). The QIF is a synthesis of 25 frameworks that resulted in the identification of 14 distinct steps for quality implementation. These steps were then divided into four temporal phases, which can serve as a roadmap for implementation in community settings such as schools. The process revealed considerable agreement among existing frameworks, thus presenting a good starting point for the integration of implementation science with CSPAP strategies. Briefly, the phases of the QIF include the following: (1) initial considerations regarding the host setting, (2) creating a structure for implementation, (3) ongoing structure once implementation begins, and (4) improving future applications. The 14 critical steps are nested within these phases and are outlined in the context of BAC in Table 1. For a more detailed description of the QIF and its application, see Meyers et al. (22, 23).

**Table 1:** Quality Implementation Framework (QIF) phases and critical steps with examples from Be a Champion! (BAC) implementation.

QIF	Example actions from BAC implementation
**Phase I: Initial considerations regarding the host setting**	
1. Needs and resources assessment	Principals completed a comprehensive school physical activity program (CSPAP) specific assessment of their school systems, policies, practices, and environments. Champion teams independently completed the School Physical Activity Policy Assessment and Physical Activity Resource Assessment
2. Fit assessment	The design of BAC was for each school to develop an action plan tailored to the results of the needs and resource assessment. As such, results of the assessment were shared with the champion teams mapped to specific activities for them to consider adding to their action plans
3. Capacity/readiness assessment	Principals and champions completed thee needs and resource assessment. All school staff and teachers completed a brief, online readiness and capacity survey. Results were shared with the champion teams
4. Possibility for adaptation	As the implementation of BAC varies with the needs and desires of the champion teams who choose from a menu of evidence-based strategies
5. Buy-in; supportive climate	To determine buy-in from other school faculty and staff, BAC conducted electronic school-wide surveys to answer questions related to knowledge, skills, attitudes, toward physical activity (PA) efforts in their schools
6. General org. capacity building	General organization capacity was addressed through the provision of tools and resources to the champion team to implement the strategies identified in the school action plan
7. Staff recruitment/maintenance	Champions were selected by principals, who chose individuals with a close personal or professional connection to PA in the school setting. Champion team members were selected by the champion in consultation with the principal with guidance from the research team liaison
8. Pre-innovation training	The initial in-service training was conducted to present champions with the research team’s intended timeline and actions. Using a modified version of a Director of Physical Activity certification, champions were given information on preparing for CSPAP implementation in their schools. A training manual was also developed and provided, which included an overview of CSPAP, example assessment tools, as well as strategies for implementation, goal setting, and action planning

**Phase II: Structure for implementation**	
9. Implementation teams	Implementation teams consisted of the selected champion and two to three other members chosen by the champion from school teachers or staff. In BAC, members came from the school wellness councils
10. Implementation plan	After each in-service training, implementation team leaders (champions) were given tasks to work on assessing their schools, goal setting, and action planning. The action plan took the form of an implementation plan, with activities and milestones

**Phase III: Ongoing support strategies**	
11. TA/coaching/supervision	After training on goal setting and action planning, champions had full access to the research team so that any questions or concerns could be addressed and were in regular contact for technical assistance
12. Process evaluation	To monitor the implementation of BAC, systematic observations were conducted across all sectors of the school day (e.g., before and after school, physical education, recess, and classroom). These observations over the course of a full school year to provide a comparison of student activity before and after the implementation of program components
13. Feedback mechanism	Process data were shared informally with champions, and a concise report was created for school stakeholders containing information about the initial assessments, action plans and activities, implemented components, and information on student activity based on systematic observation and accelerometer data

**Phase IV: Improving future applications**	
14. Learning from experience	Conversations with school officials were conducted to share lessons learned and plan for future implementation of current and aspirational aspects of the school action plans

## Be a Champion!

We developed BAC, a strategy for the implementation of a CSPAP, designed to streamline planning and delivery for school practitioners and standardize evaluation for researchers. BAC was designed to be coordinated by a state-level interventionist who provides training, resources, and technical assistance. In this study, the role of the interventionist was assumed by a liaison to the research team. Local implementation is to be accomplished in schools by PA Champions in coordination with the interventionist to assess, plan, implement, and monitor the CSPAP. BAC is not intended to replace existing CSPAP initiatives (e.g., The Alliance for a Healthier Generation’s Healthy Schools Program), but rather to guide the implementation of their elements.

Be a Champion! implementation includes provision of a resource toolkit, training workshops, and technical assistance. The first workshop, delivered to the CSPAP Champion of each school, introduces the CDC CSPAP guide (24), Champion team member recruitment, school needs assessment, and an introduction to action planning. Following the training, the Champions recruit their implementation team and conduct a systematic school-wide assessment to identify prospects for expansion of PA opportunities and potential challenges to successful implementation. A second workshop is conducted with the implementation team to facilitate the identification of targets for new PA opportunities. At this workshop, the interventionist presents a personalized report of the school needs assessment that highlights the opportunities uncovered by the implementation team. This information is used to develop a “menu” of EBPs tailored to the opportunities and resources of the school. The implementation team then engages in action planning to identify policy, environment, and programmatic approaches to implement. Once opportunities have been identified, a tailored action plan is developed in consideration of the desires of school personnel and available resources. Following the second training, the team implements the action plan with technical assistance provided by the state-level interventionist. We feel that an incremental, segmented approach is preferable to an “all at once” workshop approach that is often incongruent with the complexity of the school “system” and manner through which change is diffused through this complex system.

### A Case Example of BAC

Be a Champion! was implemented in three elementary schools from a rural school district in South Carolina. The activities reported here are from a larger cluster randomized trial, and all activities were approved by the Institutional Review Board of principal investigator’s institution. BAC was implemented along the four phases of the QIF include the following: (1) initial considerations regarding the host setting, (2) creating a structure for implementation, (3) ongoing structure once implementation begins, and (4) improving future applications. Table 1 presents example activities for each of the 14 critical steps of the QIF. Briefly, the first phase consisted of a comprehensive assessment of the school policy and physical environment, current school practices, of teacher and staff knowledge, and stakeholder attitudes toward CSPAP activities and goals. Phase II consisted of the identification of champions who led the efforts in the schools, which were facilitated by action plans created by the team in coordination with the research team. Action plans were tailored to the needs and resources of the school and prioritized by the champion teams based upon feedback from school stakeholders. Phase III consisted process evaluation accomplished *via* direct observation using standardized, validated form, and technical assistance from the research team liaison, which utilized subjective feedback based upon the direct observations. Phase IV was limited to conversations with school officials where lessons learned were shared, and plans for future implementation of current and aspirational aspects of the school action plans were discussed.

## Lessons Learned from BAC

A number of lessons were learned during Phase I of the QIF that informed BAC. Physical characteristics of the schools (e.g., indoor activity space) varied considerably, as would be expected, and the lack of resources in some schools (e.g., no space for before school activities) affected later action planning. The assessments suggested that school officials, staff, and teachers are generally aware and supportive of CSPAP goals and activities, but competing interests exert a greater pull than aspirations to increase youth PA. Addressing ever-changing student learning objectives in preparation for standardized testing was priority, and the limited teacher utilization of classroom brain breaks as a form of *PA during school* was clearly reflected by this prioritization. In our opinion, teachers are often reluctant to provide these breaks because they feel as if it is a misuse of instructional time, or from fear of creating chaos in their classes. Thus, this has created a need to develop and introduce more academically integrated PA breaks. Related, champion selection should be carefully considered during *Phase II*. While prevailing wisdom is often that PE teachers should serve as the champions, our experience suggest that non-PE teacher champions (e.g., library science teachers) might present advantages, as they are more aware of the challenges and priorities of other classroom teachers and might have more influence (25). Despite this observation, implementation of the assessment phase appeared to run smoothly with our existing teams. Similarly, *Phase III* appeared to be well received by the implementation teams and the champions. The training sessions were effective in supporting the teams in their action planning, and the action plans included multiple evidence-based strategies. Technical assistance was regularly utilized but was usually initiated during checkup calls rather than being initiated by the implementation teams. Process data collection was considerably time consuming, but produced actionable opportunities for quality improvement, which was often considered actionable by the champions. Overall, *Phase III* was successfully implemented, but the frequency of technical assistance and action plan implementation represents areas for improvement. Interestingly, classroom-based PA was consistently a target in the action plans despite potential resistance from classroom teachers. However, this resistance might explain the less than optimal implementation of the action plans.

## Lessons Learned from Implementing the CSPAP

*Before and after school activities* have the potential to allow students to quality minutes of PA outside of classroom breaks, physical education, and recess. Unfortunately, not all of the schools had a formal morning PA program that allowed larger numbers of students to be involved. In the case of afterschool programs, the primary interest emphasized academics with less focus on PA components. Students primarily engaged in free play with minimal program volunteer involvement. We feel that volunteer intentions were well meaning, with the best interest of the child in mind, but they had few specific policies that dictated the number of minutes and types of physical activities that needed to be offered.

*Community engagement*, one of the most challenging and infrequently studied (8), was the lowest prioritized CSPAP component and was not reflected in any of the three school implementation plans. While parents and community were encouraged to be actively involved in school events, it was a challenge for the schools to gain support outside of those participants that are always present. Shared use agreements did allow for community and school collaborations, however. Moving forward, in an area where there are numerous uncontrollable factors that affect adolescent health, we feel that there needs to be continued work in integrating lessons taught at the school with family and community input.

Overall, each school has its own intricacies that require attention to the overall environment/resources, staff involvement, and general attitudes toward health and PA. It is important to approach each school, principal, and staff member, where they are in their process of implementing CSPAP components. While we feel that each school advocated for the healthiest environment for their students, consistent program involvement varied from school to school. Some schools took a more innovative and proactive approach to wellness, while others preferred long-standing activities they were familiar with. In our opinion, the most important lesson gained is that truly understanding barriers, strengths, resources, and perceived benefits are at the forefront of gaining momentum in creating changes in current PA policies.

## Conclusion

Built upon implementation science, active living research, and the work of physical educators and other practitioners, we feel that BAC provides an innovative approach to guide the implementation of a CSPAP in low-resource elementary school settings. BAC is a promising implementation framework to support national recommendations for CSPAPs. This framework was designed to acknowledge the complex nature of school settings and the challenges faced by school professionals when attempting to implement a CSPAP. If proven acceptable, feasible, and effective, we believe that BAC! can aid practitioners and researchers in their mission to increase PA in youth across the country.

## Author Contributions

JM, RC, CW, RP, MB, DC, and AB contributed to the conceptualization and design of the study. JM, RC, and CS drafted the manuscript. RC, CW, RP, MB, DC, and AB provided a critical review and suggestions for revision of the penultimate draft.

## Conflict of Interest Statement

The authors declare that the research was conducted in the absence of any commercial or financial relationships that could be construed as a potential conflict of interest.

## Footnotes

^1^http://www.letsmoveschools.org/.

^2^http://www.shapeamerica.org/cspap/.
